# Clinical Characteristics and Risk Factors for Severe Exacerbation in Never-Smokers with Chronic Obstructive Pulmonary Disease: A Retrospective Cohort Study

**DOI:** 10.3390/healthcare13182374

**Published:** 2025-09-22

**Authors:** Josep Montserrat-Capdevila, Pilar Vaqué Castilla, Yoseba Cánovas Zaldúa, Francesc Alòs, Joan Deniel-Rosanas, Pere Simonet, Pau Olivares-Sanzo, Jennyfer Jiménez Díaz, Sandra Moreno Garcia, Araceli Fuentes, Eugeni Paredes, Pere Godoy

**Affiliations:** 1 Teaching Unit of Family and Community Care, Primary Care and Community Health, Catalan Health Institute (ICS), 25007 Lleida, Spain; 2 Primary and Community Health Care Management (GAPiC), Catalan Health Institute (ICS), 25007 Lleida, Spain; 3 Primary Care Center Passeig de Sant Joan, Catalan Health Institute (ICS), 08035 Barcelona, Spain; ycanovas.bcn.ics@gencat.cat (Y.C.Z.);; 4 Family Phsician, Executive Board of the Catalan Society of Family and Community Medicine (CAMFiC), 08009 Barcelona, Spain; joandeniel@camfic.org; 5 Primary Care Center Maria Bernades-Viladecans, Catalan Health Institute (ICS), 08840 Barcelona, Spain; 6 Clinical Assessment Unit, Primary and Community Health Care Management (GAPiC), Catalan Health Institute (ICS), 25007 Lleida, Spain; 7 Primary Care Center Onze Setembre, Catalan Health Institute (ICS), 25005 Lleida, Spain; 8 Biomedical Research Institute of Lleida (IRBLleida), University of Lleida, 25198 Lleida, Spain; pere.godoy@gencat.cat; 9 Consortium for Biomedical Research in Epidemiology and Public Health (CIBERESP), 28029 Madrid, Spain; 10 Population Cancer Registry, University Hospital of Santa Maria, 25198 Lleida, Spain

**Keywords:** COPD, never smokers, comorbidities, hospitalization, mortality, primary care

## Abstract

**Background:** Chronic obstructive pulmonary disease (COPD) in nonsmokers is increasingly recognized, yet its clinical profile and outcomes remain less well defined compared to smoking-related COPD. The aim of this study was to compare the clinical characteristics, comorbidities, and risk factors associated with severe exacerbations in nonsmoking COPD patients versus smokers. **Methods:** We conducted a prospective cohort study including 2376 patients with a diagnosis of COPD from the Lleida Health Region (Catalonia, Spain). Patients were followed for 2 years (2021–2022). Severe exacerbation was defined as hospital admission due to worsening COPD symptoms. Clinical variables were collected at baseline, and logistic regression analysis was performed to identify risk factors for severe exacerbation in the COPD-NS subgroup. **Results:** A total of 2376 COPD patients were included, of whom 966 (40.7%) were never-smokers. During the two-year follow-up, 165 patients (6.9%) required hospitalization for a severe exacerbation, nearly half of whom were never-smokers (48.5%). In multivariate analysis restricted to COPD never-smokers, the following independent predictors of hospitalization were identified: atrial fibrillation (OR: 2.35; 95% CI: 1.37–3.93), bronchiectasis (OR: 1.91; 95% CI: 1.08–3.28), and lower lung function measured by FVC (OR: 0.64; 95% CI: 0.45–0.89) and FEV1/FVC ratio (OR: 0.64; 95% CI: 0.45–0.89). Female gender was associated with a lower risk (OR: 0.44; 95% CI: 0.21–0.88). The predictive model demonstrated moderate discrimination (AUC = 0.71). **Conclusions:** COPD-NS patients represent a large proportion of the COPD population and present distinct clinical features. Although the incidence of severe exacerbation is similar to that of smokers/ex-smokers, risk factors such as atrial fibrillation and bronchiectasis have a stronger impact in this subgroup. Early identification of these factors may help guide more targeted clinical management strategies.

## 1. Background

Chronic obstructive pulmonary disease (COPD) is a major global public health issue due to its high prevalence and associated mortality [1,2,3]. Although the incidence of exacerbations appears similar among smokers and non-smokers with COPD [4], some evidence suggests that lung function decline may be worse in patients who have never smoked (COPD-NS) [5,6]. Preventing exacerbations is therefore a key strategy to improve prognosis in these patients.

Up to 50% of patients experience severe exacerbations despite optimal therapy, leading to higher mortality and impaired quality of life. COPD in never-smokers is associated with several risk factors, including indoor biomass smoke, ambient air pollution, occupational exposures, poorly controlled asthma, prior tuberculosis, and recurrent respiratory infections. COPD-NS may account for up to 40% of all COPD cases globally, although proportions vary widely across regions (2–60%) [2,7].

Air pollution and household biomass smoke represent major contributors. Ambient pollutants are strongly linked to COPD development [8,9], while biomass fuels for cooking and heating remain a key source of household air pollution in low-resource settings [10,11]. Biological mechanisms in COPD-NS differ from tobacco-related COPD: ambient pollutants activate oxidative stress and inflammation [8], biomass smoke triggers distinct cascades [12], and biomass-related COPD has been associated with altered immunity and possible autoimmune mechanisms [13,14].

Occupational exposure to vapors, gases, dusts, and fumes (VGDF) also contributes significantly [15,16,17,18,19,20], with high-risk jobs including agriculture, manufacturing, mining, and warehouse work. Epidemiological cohorts such as SAPALDIA [17] and MESA [18,19] have confirmed its impact on airflow limitation and COPD-related mortality.

Taken together, COPD-NS shows distinct epidemiology, risk factors, and comorbidities compared to smoking-related COPD. There is a pressing need for region-specific data to better understand predictors of severe exacerbations and to guide tailored interventions that may improve prognosis and quality of life [6,7].

## 2. Methods

We conducted a retrospective cohort study between 2021 and 2022 involving 2376 patients with a confirmed diagnosis of COPD receiving care through the Catalan Health Service (Institut Català de la Salut, ICS) in the Lleida Health Region, Spain, which serves a population of approximately 120,166 inhabitants. All participants provided written informed consent, and the study protocol was approved by the Clinical Research Ethics Committee of the Primary Care Research Institute (IDIAP) Jordi Gol in Barcelona (reference 22/242-P).

Inclusion criteria were patients aged > 40 years with a recorded diagnosis of COPD according to the Global Initiative for Chronic Obstructive Lung Disease (GOLD) 2020 criteria in their electronic health records (eCAP system). All patients had undergone spirometry testing within the four years prior to study enrollment.

Exclusion criteria were patients with a clinical diagnosis of COPD in the electronic health record were excluded if the available spirometry was not compatible with COPD, or if the most recent spirometry had been performed more than four years before the index date.

At baseline, the following variables were collected for each participant: age, sex, smoking status, COPD severity, and comorbidities.

The primary outcome was hospital admission for COPD exacerbation, obtained from the minimum basic hospital discharge dataset from all referral hospitals in the region. A COPD exacerbation was defined as an acute worsening of respiratory symptoms—specifically, increased dyspnea and/or increased sputum volume and purulence—requiring hospitalization. For each patient, the number of hospital admissions due to exacerbation between 1 January 2021, and 31 December 2022, was recorded.

### Statistical Analysis

Descriptive statistics were calculated. Quantitative variables were summarized using means and standard deviations, while categorical variables were presented as absolute and relative frequencies. To assess associations between independent variables and the clinical outcome (hospitalized exacerbation), we used the Chi-square test for categorical variables and the Student’s *t*-test or Mann–Whitney U test for continuous variables, as appropriate.

Crude odds ratios (ORs) were estimated for each independent variable. The dataset was randomly divided into a derivation cohort (70%) and a validation cohort (30%). A predictive score was developed using the derivation cohort and evaluated in the validation cohort, where its predictive capacity (discrimination and calibration) was assessed. The score included all variables significantly associated with the outcome (*p* < 0.05).

All statistical analyses were performed using SPSS software, version 29.0.1.0 (SPSS Inc., Chicago, IL, USA).

## 3. Results

A total of 2376 COPD patients were included and followed for two years, of whom 966 (40.7%) were never-smokers. Baseline characteristics are summarized in Table 1. Overall, 20.6% of COPD patients were female, with a mean age of 71 years. Compared to smokers/ex-smokers, COPD never-smokers were significantly older (mean age: 78 vs. 71 years, *p* < 0.05), had a higher proportion of females (23% vs. 19%, *p* < 0.05), and had worse lung function as measured by FEV1 (1.60 L vs. 1.86 L, *p* < 0.05). Never-smokers also showed a higher prevalence of comorbidities, particularly arterial hypertension (57.4%) and bronchiectasis (17.1%), and were more frequently diagnosed with COVID-19 prior to enrollment (36.1% vs. 26.5%).

During follow-up, 165 patients (6.9%) required at least one hospitalization due to severe COPD exacerbation, of whom 80 (48.5%) were never-smokers (Table 2). Compared to hospitalized smokers/ex-smokers (n = 85), hospitalized never-smokers were older (81 vs. 69 years, *p* < 0.05) and had a higher burden of comorbidities. The most relevant differences included atrial fibrillation (32.5% vs. 12.9%, *p* < 0.05), arterial hypertension (73.8% vs. 49.4%, *p* < 0.05), chronic kidney disease (20.0% vs. 8.2%, *p* < 0.05), and bronchiectasis (26.7% vs. 21.2%).

Among never-smokers, those hospitalized for severe exacerbations had significantly lower lung function (FEV1: 1.23 L vs. 1.35 L, *p* < 0.05; FEV1/FVC: 0.58 vs. 0.62, *p* < 0.05), as well as a higher prevalence of bronchiectasis (26.2% vs. 16.3%) and comorbidities such as atrial fibrillation, ischemic heart disease, heart failure, chronic kidney disease, and dementia (Table 3). They also more frequently required domiciliary oxygen therapy.

Descriptive statistics were presented as means with standard deviations (SDs) for continuous variables and as frequencies with percentages for categorical variables. Comparisons between groups were performed using the Student’s *t* test or Mann–Whitney *U* test for continuous variables and the Chi-square test or Fisher’s exact test for categorical variables, as appropriate.

To identify independent predictors of hospitalization for severe COPD exacerbation among never-smokers, we performed a multivariate logistic regression analysis. Variables with a *p* value < 0.10 in the univariate analysis, as well as clinically relevant covariates, were considered for inclusion in the model. A stepwise backward selection procedure was applied to obtain the final model, while avoiding multicollinearity.

The following confounders were evaluated: age, sex, body mass index (BMI), smoking exposure (passive or former), cardiovascular comorbidities (hypertension, ischemic heart disease, atrial fibrillation, and heart failure), metabolic comorbidities (diabetes mellitus and dyslipidemia), respiratory comorbidities (asthma, bronchiectasis, and obstructive sleep apnea), and lung function parameters (FEV1, FVC, and FEV1/FVC ratio). Adjusted odds ratios (ORs) with 95% confidence intervals (CIs) were calculated (Table 4).

**Figure 1:**
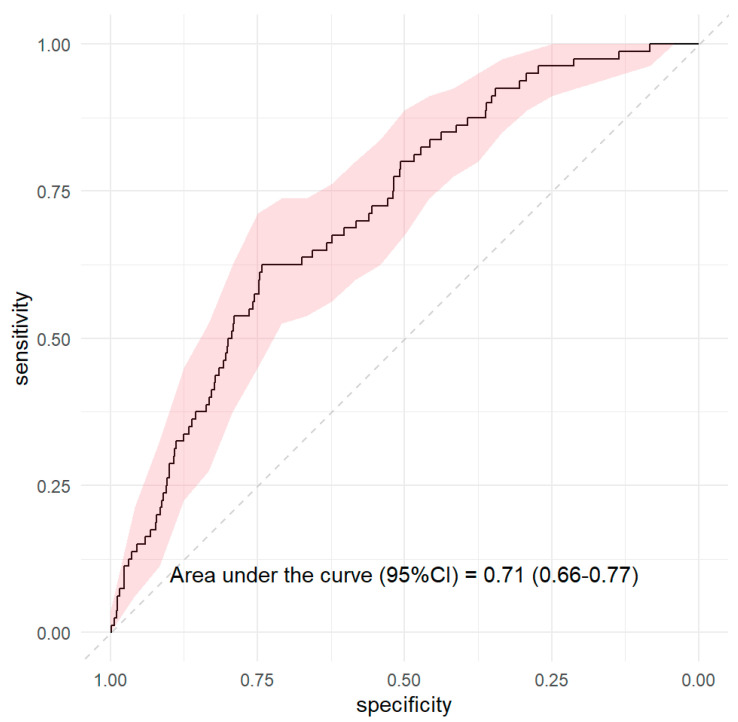
ROC curve: area under the curve for severe exacerbation in COPD-NS.

Model performance was assessed by discrimination using the area under the receiver operating characteristic curve (AUC) (Figure 1). Statistical significance was set at a two-tailed *p* value < 0.05. All analyses were conducted using SPSS.

## 4. Discussion

In this study, 40.7% of COPD patients were never-smokers, reflecting the heterogeneity reported across different populations. The prevalence of COPD in never-smokers (COPD-NS) ranges widely, from 2.1% in the Spanish population according to Miravitlles et al. [21] to as high as 58.8% in other cohorts [22]. Consistent with prior studies [18,23,24,25,26,27], COPD-NS patients were older compared to smokers and ex-smokers. Lamprecht et al. reported an increasing prevalence of moderate-to-severe COPD-NS with advancing age [23], and similar correlations have been found by Hagstad et al. [24]. This may relate to prolonged exposure to biomass smoke and indoor pollution, which require extended durations before disease manifestation and progression [25,28].

The proportion of women among COPD-NS patients was higher (23% vs. 19%), consistent with findings from other studies [18,21,23,29,30], albeit lower than the 48% reported by Lamprecht et al. Tobacco smoking, more prevalent among men, remains the principal risk factor for moderate-to-severe COPD and partially explains these gender differences. Tobacco smoke contains thousands of compounds, including free radicals and oxidants that induce chronic inflammation and apoptosis [31]. In our study, male sex was independently associated with an increased risk of hospitalization, suggesting potential differences in phenotype, comorbidity burden, or healthcare-seeking behavior that deserve further exploration [32].

Contrary to the general perception that airflow limitation in COPD-NS is mild (GOLD stage 1) [7], our study found that COPD severity (measured by FEV1) was worse in never-smokers than in smokers/ex-smokers. Underdiagnosis and underreporting in primary care databases may contribute to this discrepancy, as less symptomatic non-smokers might be less likely to be diagnosed [6]. Ojuawo et al. reported that 50% of COPD-NS patients had moderate-to-very-severe disease (GOLD 2–4), with 23.7% in GOLD 4 [30]. The greater comorbidity burden observed in COPD-NS, particularly cardiovascular disease, may contribute to functional limitations, reduced quality of life, and poorer outcomes, as previously reported [7,30].

Among those hospitalized for severe COPD exacerbations during the two-year follow-up, 48.5% were never-smokers. Despite usually milder disease in COPD-NS, the high hospitalization rate may be attributed to greater comorbidity and a higher prevalence of bronchiectasis (17.1% vs. 8.6%). The PLATINO study similarly reported a higher incidence of hospitalization for exacerbation in non-smokers, with risks up to three times greater (OR: 2.92; 95% CI: 1.21–7.07) [27,30]. However, data across international cohorts are not uniform, as illustrated by the Korean KOCOSS study, which reported no differences in exacerbation rates according to smoking status [33].

Within COPD-NS, hospitalized patients were older, had worse lung function, and had higher comorbidity, especially hypertension and atrial fibrillation, mirroring risk factors seen in smokers/ex-smokers [34]. The role of cardiovascular comorbidity as a predictor of hospitalization and mortality in COPD has been reported in other studies [35,36].

Our multivariate analysis identified male gender, older age, bronchiectasis, and atrial fibrillation as independent risk factors for hospitalization in COPD-NS. The relationship between COPD and atrial fibrillation is clinically relevant, as COPD is frequently complicated by arrhythmias through mechanisms such as hypoxemia [37], acidosis [38], and cor pulmonale [39], and its incidence has been shown to be inversely related to lung function [40].

Bronchiectasis nearly doubled the risk of severe exacerbations in COPD-NS. This association may be mediated by increased bronchial infection with potentially pathogenic microorganisms [41]. Specifically, humidity and fungal colonization have been consistently linked with infections in COPD-NS (OR: 1.50; 95% CI: 1.32–1.70) [41].

## 5. Limitations

This study may be limited by potential under-registration of variables in primary care databases and in spirometry measurements conducted by different healthcare professionals. Nevertheless, all spirometries were performed by trained personnel using the same brand and model of spirometer, mitigating some concerns regarding measurement consistency. Additionally, we acknowledge that environmental and occupational exposures were not captured in our dataset, and underdiagnosis in primary care remains a potential limitation influencing prevalence and severity estimates.

## 6. Conclusions

The prevalence of COPD in never-smokers (COPD-NS) is substantial. This study reinforces that COPD-NS is not a benign condition, but rather a clinically relevant phenotype with significant morbidity. Although COPD in smokers/ex-smokers and never-smokers shares some common features, distinct risk factors exist for each subgroup. The proportion of severe exacerbations requiring hospitalization in COPD-NS is comparable to that in smokers/ex-smokers. However, the presence of bronchiectasis, atrial fibrillation, advanced age, and male sex are significant risk factors in COPD-NS. Clinicians should consider these factors to identify high-risk patients, optimize individualized management, and implement closer follow-up strategies aimed at preventing hospitalizations and improving quality of life and survival in COPD-NS patients.

## Figures and Tables

**Table 1:** Baseline characteristics of COPD never-smokers vs. smokers.

	COPD (n = 2376)	COPD-NS (n = 966)	COPD Smokers/Ex-Smokers (n = 1410)	*p* *
Age (years), mean (SD)	71.0 (63.0–78.0)	78.0 (72.0–84.0)	66.0 (59.0–73.0)	<0.001
Gender (female)	490 (20.6%)	222 (23.0%)	268 (19.0%)	0.021
Alcohol consumption Non-alcohol dependency	2240 (94.3%) 58 (2.4%)	943 (97.6%) 8 (0.8%)	1297 (92.0%) 50 (3.6%)	<0.001
FVC, mean (SD)	2.97 (2.36–3.60)	2.71 (2.10–3.31)	3.12 (2.56–3.76)	<0.001
FEV1, mean (SD)	1.75 (1.34–2.19)	1.60 (1.12–2.03)	1.86 (1.44–2.30)	<0.001
FEV1/FVC, mean (SD)	0.62 (0.54–0.66)	0.62 (0.55–0.66)	0.61 (0.54–0.66)	0.095
AFib	276 (11.6%)	172 (17.8%)	104 (7.38%)	<0.001
Anaemia	194 (8.2%)	103 (10.7%)	104 (7.4%)	<0.001
Antecedent of cancer	477 (20.1%)	250 (25.9%)	227 (16.1%)	<0.001
Ictus	42 (1.8%)	20 (2.1%)	22 (1.6%)	0.442
Ischemic cardiopathy	141 (5.9%)	81 (8.4%)	60 (4.3%)	<0.001
Cardiac heart failure	145 (6.1%)	97 (10.0%)	48 (3.4%)	<0.001
Chronic kidney disease	300 (12.6%)	177 (18.3%)	123 (8.7%)	<0.001
Chronic hepatopathy	11 (0.4%)	0 (0.0%)	11 (0.8%)	0.004
Bronchiectasis	286 (12.0%)	165 (17.1%)	121 (8.6%)	<0.001
Cognitive deterioration	53 (2.2%)	33 (3.4%)	20 (1.4%)	0.002
Dementia	55 (2.3%)	33 (3.4%)	22 (1.6%)	0.005
Arterial hypertension	1365 (57.4%)	645 (66.8%)	720 (51.1%)	<0.001
Dyslipidemia	1009 (42.5%)	403 (41.7%)	606 (43.0%)	0.570
Type 2 diabetes mellitus	619 (26.1%)	264 (27.3%)	355 (25.2%)	0.260
Obstructive sleep apnea	159 (6.7%)	77 (7.8%)	82 (5.8%)	0.048
Antecedents of SARS-CoV-2 infection	722 (30.4%)	349 (36.1%)	373 (26.5%)	<0.001
Domiciliary oxygen therapy	6 (0.3%)	5 (0.5%)	1 (0.1%)	0.044
Patient dependency	85 (3.6%)	43 (4.5%)	42 (3.0%)	0.074
Residential care	31 (1.3%)	14 (1.5%)	17 (1.2%)	0.741

* X2 test; SD, standard deviation; *FVC*, forced vital capacity; *FEV1*, forced expiration volume in 1 s; *AFib*, atrial fibrillation.

**Table 2:** Characteristics of hospitalized COPD patients.

	COPD (n = 165)	COPD-NS (n = 80)	COPD Smokers/Ex-Smokers (n = 85)	*p* *
Age (years), mean (SD)	75.0 (67.0–82.0)	81.0 (75.8–85.0)	69.0 (63.0–75.0)	<0.001
Gender (female)	30 (18.2%)	13 (16.2%)	17 (20.0%)	0.673
Alcohol consumption Non-alcohol dependency	158 (95.8%) 3 (1.8%)	80 (100%) 0 (0.0%)	78 (91.8%) 3 (3.5%)	0.030
FVC, mean (SD)	2.65 (2.13–3.20)	2.45 (2.02–2.92)	2.84 (2.28–3.36)	0.008
FEV1, mean (SD)	1.46 (1.17–1.82)	1.35 (1.09–1.68)	1.49 (1.21–1.92)	0.066
FEV1/FVC, mean (SD)	0.57 (0.51–0.64)	0.58 (0.52–0.64)	0.57 (0.49–0.62)	0.237
AFib	37 (22.4%)	26 (32.5%)	11 (12.9%)	0.005
Anaemia	22 (13.3%)	14 (17.5%)	8 (9.41%)	0.194
Antecedent of cancer	43 (26.1%)	23 (28.7%)	20 (23.5%)	0.558
Ictus	2 (1.21%)	2 (2.50%)	0 (0.0%)	0.234
Ischemic cardiopathy	14 (8.48%)	8 (10.0%)	6 (7.06%)	0.691
Cardiac heart failure	19 (11.5%)	13 (16.2%)	6 (7.1%)	0.109
Chronic kidney disease	23 (13.9%)	16 (20.0%)	7 (8.2%)	0.051
Chronic hepatopathy	1 (0.61%)	0 (0.00%)	1 (1.18%)	1.000
Bronchiectasis	39 (23.6%)	21 (26.2%)	18 (21.2%)	0.560
Cognitive deterioration	7 (4.24%)	3 (3.75%)	4 (4.71%)	1.000
Dementia	5 (3.03%)	4 (5.00%)	1 (1.18%)	0.200
Arterial hypertension	37 (22.4%)	26 (32.5%)	11 (12.9%)	0.005
Dyslipidemia	49 (29.7%)	19 (23.8%)	30 (35.3%)	0.147
Type 2 diabetes mellitus	619 (26.1%)	264 (27.3%)	355 (25.2%)	0.260
Obstructive sleep apnea	9 (5.45%)	5 (6.25%)	4 (4.71%)	0.741
Antecedents of SARS-CoV-2 infection	75 (45.5%)	42 (52.5%)	33 (38.8%)	0.108
Domiciliary oxygen therapy	2 (1.21%)	2 (2.50%)	0 (0.00%)	0.234
Patient dependency	11 (6.67%)	6 (7.50%)	5 (5.88%)	0.917
Residential care	2 (1.21%)	1 (1.25%)	1 (1.89%)	1.00

* X2 test; *SD*, standard deviation; *FVC*, forced vital capacity; *FEV1*, forced expiration volume in 1 s; *AFib*, atrial fibrillation.

**Table 3:** Hospitalized vs. non-hospitalized COPD-NS patients.

	COPD-NS Patients Who Were Not Hospitalized for Severe Exacerbation of COPD (N = 886)	COPD-NS Patients Hospitalized for COPD Exacerbation Hospitalized (N = 80)	*p* *
Age (years), mean (SD)	77.0 (71.0–84.0)	81.0 (75.8–85.0)	0.003
Gender (female)	209 (23.6%)	13 (16.2%)	0.175
Alcohol consumption Non-alcohol dependency	863 (97.4%) 8 (0.9%)	80 (100.0%) 50 (0.0%)	0.679
FVC, mean (SD)	2.74 (2.12–3.36)	2.45 (2.02–2.92)	0.011
FEV1, mean (SD)	1.62 (1.23–2.06)	1.35 (1.09–1.68)	<0.001
FEV1/FVC, mean (SD)	0.62 (0.56–0.66)	0.58 (0.52–0.64)	0.003
AFib	146 (16.5%)	26 (32.5%)	0.001
Anaemia	89 (10.0%)	14 (17.5%)	0.060
Antecedent of cancer	227 (25.6%)	23 (28.7%)	0.632
Ictus	18 (2.03%)	2 (2.50%)	0.678
Ischemic cardiopathy	81 (8.4%)	60 (4.3%)	<0.001
Cardiac heart failure	97 (10.0%)	48 (3.4%)	<0.001
Chronic kidney disease	177 (18.3%)	123 (8.7%)	<0.001
Chronic hepatopathy	0 (0.0%)	11 (0.8%)	0.004
Bronchiectasis	144 (16.3%)	21 (26.2%)	0.034
Cognitive deterioration	30 (3.39%)	3 (3.75%)	0.749
Dementia	29 (3.27%)	4 (5.00%)	0.344
Arterial hypertension	586 (66.1%)	59 (73.8%)	0.208
Dyslipidemia	370 (41.8%)	33 (41.2%)	1.000
Type 2 diabetes mellitus	245 (27.7%)	19 (23.8%)	0.536
Obstructive sleep apnea	72 (8.13%)	5 (6.25%)	0.705
Domiciliary oxygen therapy	3 (0.34%)	2 (2.50%)	0.057
Patient dependency	37 (4.18%)	6 (7.50%)	0.159
Residential care	13 (1.47%)	1 (1.25%)	1.000

* X2 test; *SD*, standard deviation; *FVC*, forced vital capacity; *FEV1*, forced expiration volume in 1 s; *AFib*, atrial fibrillation.

**Table 4:** Multivariate logistic regression in COPD-NS.

	Odds Ratio	CI 2.5	CI 97.5	*p*
Age (years)	1.03009169	0.998768561	1.0638472	0.065
Gender (female)	0.44341355	0.210548594	0.8813557	0.025
FVC	0.63754613	0.447821617	0.8860340	0.010
FEV1/FVC	0.02236115	0.001817417	0.2986086	0.003
AFib	2.3486499	1.370358/188	3.9346937	0.001
Bronchiectasis	1.91330570	1.083618008	3.2770213	0.021

*FVC*, forced vital capacity; *FEV1*, forced expiration volume in 1 s; *AFib*, atrial fibrillation.

## Data Availability

The original contributions presented in this study are included in the article. Further inquiries can be directed to the corresponding author.
